# Qualitative evaluation of the Systems Analysis and Improvement Approach as a strategy to increase HIV testing in family planning clinics using the Consolidated Framework for Implementation Research and the Implementation Outcomes Framework

**DOI:** 10.1186/s43058-022-00342-x

**Published:** 2022-09-08

**Authors:** McKenna C. Eastment, Jessica E. Long, George Wanje, Barbra A. Richardson, Emily Mwaringa, Kenneth Sherr, Ruanne V. Barnabas, Kishorchandra Mandaliya, Walter Jaoko, R. Scott McClelland

**Affiliations:** 1 Department of Medicine, University of Washington, 325 9th Avenue, Box 359909, Seattle, WA 98104 USA; 2 Department of Epidemiology, University of Washington, Seattle, WA USA; 3 Department of Global Health, University of Washington, Seattle, WA USA; 4 Department of Biostatistics, University of Washington, Seattle, WA USA; 5 Vaccine and Infectious Disease Division, Fred Hutchinson Cancer Research Center, Seattle, WA USA; 6 Mombasa County Department of Health, Mombasa, Kenya; 7 Department of Industrial & Systems Engineering, University of Washington, Seattle, WA USA; 8 Division of Infectious Diseases, Massachusetts General Hospital, Boston, MA USA; 9 Harvard Medical School, Boston, MA USA; 10 Department of Medical Microbiology and Immunology, University of Nairobi, Nairobi, Kenya

**Keywords:** SAIA, Systems Analysis and Improvement Approach, Consolidated Framework for Implementation Research, Implementation Outcomes Framework, Evaluation, Implementation strategy, HIV testing, Family planning, Systems engineering, Mechanisms of action

## Abstract

**Background:**

Significant gaps remain in HIV testing and counseling (HTC) in family planning (FP) clinics. To address these gaps, our group tested an implementation strategy called the Systems Analysis and Improvement Approach (SAIA), an evidenced-based multi-component implementation strategy focused on improving entire care cascades. In a cluster randomized trial of 24 FP clinics in Mombasa County, Kenya, SAIA led to a significant increase in HTC in intervention clinics compared to control clinics. The objective of this manuscript was to evaluate SAIA using the Consolidated Framework for Implementation Research (CFIR) and assess the Implementation Outcomes Framework outcomes of acceptability, appropriateness, and feasibility.

**Methods:**

This qualitative assessment was nested within the cluster-randomized trial. Data collection included questionnaires to assess modifiable and non-modifiable health system factors related to HTC and in-depth interviews to query clinic norms, priorities, communication strategies, and readiness for change. The primary outcomes of interest were feasibility, appropriateness, and acceptability of SAIA. Data on inner setting and structural characteristics of FP clinics were collected to inform how context may impact outcomes. All interviews were recorded and analyzed using a rapid assessment approach*.*

**Results:**

Of the 12 intervention clinics, 6 (50%) were public facilities. Availability of resources varied by clinic. Most clinics had a positive implementation climate, engaged leadership, and access to resources and information. While not all clinics identified HTC as a clinic priority, most reported a strong culture of embracing change and recognition of the importance of HIV testing within FP clinics. Interviews highlighted very high acceptability, appropriateness, and feasibility of SAIA. The implementation strategy was not complicated and fit well into existing clinic processes. In particular, staff appreciated that SAIA allowed clinic staff to generate contextually relevant solutions that they implemented.

**Conclusions:**

SAIA was implemented in FP clinics of varying sizes, capacity, and management support and was found to be acceptable, appropriate, and feasible. The agency that clinic staff felt in proposing and implementing their own solutions was likely part of SAIA’s success. We anticipate this will continue to be a mechanism of SAIA’s success when it is scaled up to more clinics in future trials.

**Trial registration:**

ClinicalTrials.gov (NCT02994355) registered 16 December 2016.

Contributions to the literature

- The Systems Analysis and Improvement Approach (SAIA) is an evidenced-based multi-component implementation strategy that has been used to improve care cascades.
- Little is known about the mechanisms of action through which SAIA works to impact care delivery.
- We used qualitative assessments nested within a cluster-randomized trial using SAIA to improve uptake of HIV testing and counselling in Mombasa County, Kenya.
- SAIA was found to be acceptable, appropriate, and feasible. Successful implementation was facilitated by clear communication, well-defined goals, and low complexity.
- The evidence of mechanisms through which SAIA operates can inform future SAIA applications.

## Introduction

To target the first UNAIDS 95–95-95 goal of 95% of people being aware of their HIV status, strategies to integrate HIV testing and counseling (HTC) into other service delivery points offer great potential. In Kenya, women of reproductive age have the highest HIV incidence of any demographic group and are therefore a priority population for HIV testing [1]. Providing HTC services at family planning (FP) clinics is a promising strategy to reach these women, as many women in Kenya access FP services [2], and this has risen in recent years [3]. However, a recent survey of 58 FP clinics in Mombasa County, Kenya, found that only 10% of new FP clients were tested for HIV [4], demonstrating that uptake of HTC integration in FP clinics may remain low despite the potential benefit.

To address the gap between a known evidence-based intervention and its implementation in a real-world setting, we tested an implementation strategy called the Systems Analysis and Improvement Approach (SAIA) as a method of increasing HTC in FP clinics [5]. SAIA is an evidenced-based multi-component implementation strategy focused on improving entire care cascades that can be adapted to fit a variety of contexts. It was originally developed in 2012 as a package of systems engineering tools and tested as a strategy to improve performance of the prevention of mother to child transmission (PMTCT) of HIV care cascades in Mozambique, Kenya, and Cote d’Ivoire [6–9]. SAIA has five steps. Step 1 uses an Excel-based “cascade analysis” tool to quantify the number of individuals who complete each step of a process and identify priority steps for improvement [10, 11]. Step 2 involves sequential process flow mapping with clinic staff to identify modifiable bottlenecks in the system. Plan-do-study-act cycles are repeated in steps 3 through 5 [12]. Specifically, step 3 develops and implements workflow modifications (micro-interventions) to address a bottleneck identified in step 2. Step 4 assesses impact of the modification and recalculates the cascade analysis from step 1. Step 5 repeats the cycle. Our study was the first to use SAIA in family planning clinics, but this implementation strategy has also been tested to improve hypertension management [13] as well as mental health care [14] in outpatient settings in Mozambique.

In a sample of 24 FP clinics in Mombasa County, Kenya, 12 were randomized to the SAIA strategy and 12 were randomized to usual procedures [5]. SAIA led to a substantial increase in both HIV counseling and testing in intervention clinics compared to control clinics. In primary effectiveness analyses, 85% (740/868) of new FP clients received pre-HIV test counseling in intervention clinics compared to 67% (1036/1542) in control clinics (prevalence rate ratio [PRR] 1.27, 95% confidence interval [CI] 1.15–1.30). The increased pre-test counseling resulted in 42% (364/859) of FP clients being tested for HIV at intervention clinics compared to 32% (485/1521) at control clinics (prevalence rate ratio [PRR] 1.33, 95% confidence interval [CI] 1.16–1.52).

The effectiveness of SAIA as an implementation strategy provides only part of the information needed to guide decisions about future scale-up. Evaluation of implementation outcomes from the perspective of healthcare workers can provide additional information that is essential to inform wider implementation. To this end, the present manuscript utilizes qualitative data, questionnaires, and validated survey instruments collected in parallel with the trial to address two objectives. First, we evaluated SAIA as a strategy to improve HIV counseling and testing using the Consolidated Framework for Implementation Research (CFIR) to examine inner setting and intervention characteristics [15, 16]. Second, we assessed SAIA’s acceptability, appropriateness, and feasibility from Proctor’s Implementation Outcomes Framework (IOF) [17] using psychometrically validated tools developed by Weiner et al. [18].

## Methods

### Study design

This qualitative assessment was nested within a cluster-randomized trial comparing use of SAIA versus usual procedures to increase HTC in FP clinics in Mombasa County, Kenya. Specific steps of this trial have been previously described [5]. Briefly, the five-step SAIA cycle was adapted to target the HTC process in FP clinics, then tested within 12 clinics compared to 12 controls. To begin the trial, study-specific training materials and a training booklet were developed, and the cascade analysis tool was adapted by study staff to collect HTC data. The input data required (number of new FP clients seen, number counseled, number known positive, number tested) were obtained from FP registers that are maintained by staff within all FP clinics as part of routine clinic data collection. After randomization, a representative from each intervention clinic was provided with the training booklet and attended a full day SAIA training led by study staff, which included training in completion of the cascade analysis tool and sequential process flow mapping. In addition, each clinic conceptualized their first micro-intervention with facilitation by study staff. Once the study began, study staff visited each intervention clinic once a month to complete the cascade analysis tool with data from the previous month (step 1) and oversee completion of the plan-do-study-act cycles (steps 3–5), which included reviewing micro-interventions from the previous month, assessing progress, and setting new micro-interventions to implement over the next cycle. The second step of SAIA, sequential process flow mapping, was repeated when clinic process or flow was significantly changed. Monthly SAIA visits were conducted in clinics and led by study staff, while clinic staff were responsible for selecting micro-interventions and implementing them within the clinic.

The study consisted of two stages. Study stage 1, conducted from December 2018 to November 2019, consisted of 12 months of SAIA cycles as described above, led by study staff, to determine the effectiveness of SAIA in increasing HTC in Mombasa County. Stage 2 was conducted from February 2020 to January 2021 and involved the same steps but was led by staff within the Mombasa County Department of Health Services (DOHS) who were trained in SAIA. The purpose of this stage was to assess if the effectiveness of SAIA could be sustained as the intervention was transitioned to DOHS leadership, with minimal support from the study staff, and embedded as part of their programmatic activities.

Data collection for the present analysis included questionnaires and interviews collected during study stage 1 and at the end of stage 2. Stage 1 data collection included a questionnaire adapted from the SAIA-SCALE study [8], which assessed SAIA to improve the PMTCT of HIV care cascade. The questionnaire assessed facility-level characteristics (e.g., clinic size, location), clinic manager characteristics (e.g., education level, experience), challenges faced by the clinic (e.g., training, supply stockouts), and infrastructure that could impact the successful implementation of HIV testing and counseling services, including availability of resources and organizational infrastructure and communication. Stage 1 also included interviews, which assessed culture, resources, and HIV testing procedures within clinics. In stage 2, exit interviews were completed among intervention clinics to assess their experience with SAIA and allow for open-ended responses about the feasibility, acceptability, and appropriateness of SAIA in their clinic. Reporting of these results abide by the Standards for Reporting Qualitative Research (SRQR) [19].

### Setting

All study clinics were located in urban and peri-urban areas within Mombasa County. As of 2018, an estimated 5.6% of Mombasa County’s 1.2 million residents were living with HIV [20]. Prevalence among women was significantly higher, at 10.5%. Kenya National Guidelines for HIV Testing and Counselling, which were first published in 2008 and revised in 2010, stipulate that all new FP clients should be offered HTC [21]. In the context of our study, counseling refers to pre-test counseling, in which care providers recommend opt-out HIV testing and ask family planning clients if they are willing to be tested. Testing refers to HIV testing offered through the FP clinic facility. As of 2018, family planning services in Mombasa were offered through approximately 170 public and private FP clinics, all of which receive free commodities, including HIV testing supplies, from the Mombasa County DOHS. Commodity distribution is regulated through monthly data collection on a paper register.

### Study participants

Questionnaires and interviews were conducted with staff from each participating FP clinic. The present analysis focuses on data collected from intervention clinics. During study stage 1, FP staff at all intervention clinics (*n* = 12) were asked to complete a verbally administered questionnaire, followed by a separate interview. At the end of stage 2, staff from all intervention clinics were asked to complete a second round of questionnaires and interviews. In both study stages, the data were purposively collected from one clinic staff member from each clinic who was familiar with the study. Most participating clinics were small with few staff members, so a specific staff member functioned as the lead on study-related activities, including interviews. The staff members who participated in this role included clinic managers, “in-charge” nurses (lead nurses), nursing staff, and lab technicians. Staff completing these assessments provided written informed consent prior to the COVID-19 pandemic. During the pandemic, data collection was completed remotely by phone and staff provided verbal assent prior to interviews.

### Data collection procedures

In stage 1, questionnaires and interviews were conducted as separate study activities at different time points. Clinic managers completed stage 1 questionnaires between March 2019 and February 2020. These questionnaires were designed to assess modifiable and non-modifiable health system factors related to HIV testing at each facility. Information was collected on the population being served by the clinic, the clinic funding, HIV testing protocols, and potential challenges to offering HIV testing. Questionnaires and interviews were in English, as this is one of the official languages of Kenya and was spoken by all health care providers. Stage 1 interviews were in-depth and followed an interview guide to probe topics regarding culture and HTC practices within clinics. Both assessments were verbally administered by study staff, recorded on paper forms, then uploaded to a secure REDCap database (ITHS, Seattle, WA) [22]. Stage 1 interviews were conducted between June and December 2019, when clinics had been implementing SAIA for approximately 6 to 12 months. Clinic staff reported on clinic norms for HTC, clinic priorities, communication, readiness for change within the organization, and resource availability within their clinic.

In stage 2, questionnaires and interviews were combined into one assessment that was administered by study staff in person or by phone. Data were recorded on paper forms that included both questionnaire responses and field notes of interview responses. Stage 2 interviews used an interview guide that allowed participants to elaborate on questionnaire responses and provide open-ended answers about how SAIA was perceived in their clinic. All intervention clinics were asked to participate in this stage 2 assessment, which focused on outcomes of SAIA implementation, including the feasibility, acceptability, and appropriateness of SAIA within their facility. Due to interruptions caused by the COVID-19 pandemic and a subsequent health care worker strike, these assessments were completed approximately six months after the close of the study, between August and September 2021.

### Outcomes

In stage 1, both the questionnaire and interviews provided data on the characteristics of each FP clinic to inform how context may impact study outcomes. This round of assessment was guided by the CFIR, and elicited responses about the inner setting at each FP clinic, including the structural characteristics, networks and communication, implementation climate, readiness for implementation, and available resources [15]. Specific items assessed in the questionnaire reflected indicators to represent how well resourced a clinic was (e.g., availability of air conditioning), existing levels of communication and organization between staff members (e.g., meeting types and frequency), and facility characteristics that are specific to provision of HTC (e.g., visual and auditory privacy to conduct HTC). These indicators were used to understand how the domains within the inner setting would impact the uptake and efficacy of SAIA in improving HTC within each clinic. The second round of questionnaires and interviews conducted in stage 2 provided data on intervention characteristics, guided by CFIR, as well as implementation outcomes guided by the IOF [17]. Intervention characteristics, including adaptability, complexity, and design quality and packaging, were assessed using open ended interview questions. Implementation outcomes included feasibility, appropriateness, and acceptability, and were assessed using a combination of 5-point Likert scale questions and open-ended interview questions. The combined quantitative and qualitative assessment used to collect data on implementation outcomes was adapted from previously validated research [18].

### Analysis

All interviews were recorded and analyzed using a rapid assessment approach [23]. First, field notes were used to develop summary memos of responses from each facility. For the stage 1 interviews, a structured codebook was created in Excel based on the CFIR constructs under evaluation. A codebook for the stage 2 interviews was similarly created based on the CFIR constructs and the IOF outcomes on feasibility, acceptability, and appropriateness. The codebooks allowed for categorization of responses to the constructs of interest, as well as a category to capture emergent themes on topics outside of elicited CFIR and IOF constructs. At the end of each interview, field notes were used to map responses to the codebook and develop summaries of each response (GW). After all interviews were coded (GW), the field notes, summary memos, and coding were reviewed by two additional researchers (JEL, MCE) to assess for concordance. The three researchers met iteratively to discuss themes and address differences in interpretation of coding until concordance was reached.

Questionnaire responses were summarized using basic descriptive statistics. Likert scale data were analyzed and reported as medians and interquartile ranges (IQR).

### Ethics

This research was approved by the Kenyatta National Hospital – University of Nairobi Ethics and Research Committee and the University of Washington Institutional Review Board. The research team for this study includes researchers from the University of Washington and the University of Nairobi with expertise in implementation science, HIV, and women’s health research in Mombasa County. All interviews were conducted by members of the research team in Mombasa, overseen by the qualitative research lead (GW) who has extensive experience conducting qualitative research related to women’s health in Mombasa County. The team also includes a member of the Mombasa County DOHS (EM) who provided key insight into context within Mombasa County during the study.

## Results

### Inner setting

Data on inner setting were collected through the stage 1 questionnaires and interviews with FP clinic staff. Clinic staff at all intervention clinics (*n* = 12) completed the questionnaire, and staff at 9 (75%) intervention clinics participated in initial in-depth interviews. All questionnaires were completed with clinic managers, and in-depth interviews were conducted with clinic managers (*n* = 3), in-charge nurses (*n* = 4), a nurse (*n* = 1), and a lab technician (*n* = 1). These data were collected to characterize the organizational structure, networks and communication, and climate at each facility and to assess facilitators and barriers to the adoption of HTC within the clinics. All constructs assessed were based on the CFIR inner setting domain.

#### Structural characteristics

Of the 12 intervention clinics, 6 (50%) were public facilities and 6 (50%) were private facilities (Table 1). Five (41.7%) clinics were supported by an NGO and one (8.3%) was supported by an academic partner. Availability of resources to promote privacy and comfort for FP clients receiving HTC varied by clinic. No FP clinic had a functioning air conditioner, which was assessed as an indicator of resource availability. The majority of clinics were able to provide complete visual privacy (8; 66.7%) and complete auditory privacy (7; 58.3%) to conduct HTC visits. The remainder of the clinics had availability of partial visual privacy where other clients or staff could see part of the client-staff interaction (4; 33.3%) and partial auditory privacy where other clients or staff could overhear part of the client-staff interaction (5; 41.7%). The clinics had a median of 0.5 (IQR 0–2) providers trained in HTC. While clinics had variable access to resources, these structural characteristics were not perceived as particular barriers to implementing HTC (Table 2).

**Table 1:** Structural characteristics of intervention clinics

	*N* (%) or median (IQR)
Facility type	
Public	6 (50%)
Private	6 (50%)
Supported by NGO	5 (41.7%)
Supported by academic partner	1 (8.3%)
Functioning air conditioner	0 (0%)
Availability of visual privacy:	
Complete visual privacy	8 (66.7%)
Partial visual privacy	4 (33.3%)
Availability of auditory privacy:	
Complete auditory privacy	7 (58.3%)
Partial auditory privacy	5 (41.7)
Providers trained in HIV counseling and testing	0.5 (0–2)
Regular management meetings were held	11 (91.7%)
Occurred monthly	6/11 (54.5%)
Occurred quarterly	5/11 (45.5%)

*Abbreviations*: *NGO* non-governmental organization, *IQR* interquartile range

**Table 2:** The inner setting and intervention characteristics that impacted HTC and the use of SAIA, assessed through interviews guided by CFIR constructs

CFIR domain	Constructs and sub-constructs	Impact	Description
**Inner setting impact on HTC**	**Structural characteristics**	None noted	Participants had mixed availability of resources to promote privacy during HTC, but none identified this as a barrier to HTC
	**Networks and communication**	Facilitator	Most clinics had regular meetings and other informal communication channels, allowing for strong communication
	**Implementation climate**		
	*Tension for change*	Mixed	Facilitator: Clinics largely identified a need for HTC and reported that they embrace change Barrier: Some clinics did not believe HTC needed to be improved, and felt that the time need to make change was a barrier
	*Relative priority*	Mixed	Facilitator: Some clinics reported that HTC was a high priority Barrier: At some clinics, HTC was not identified as one of their top priorities
	*Goals and feedback*	Facilitator	Clinics reported clear goal setting, both internally and from the DOHS. Clinics reported clear methods of tracking progress
	*Learning climate*	Mixed	Facilitator: Some clinics noted group-consensus problem-solving, which empowered staff to be involved in solution generation Barrier: Other clinics described top-down approaches, which may take more time to effect change
	**Readiness for implementation**		
	*Leadership engagement*	Mixed	Facilitator: DOHS support and resources were an important facilitator for all clinics Barrier: Clinics had mixed leadership engagement internally. Low engagement impacted ability to obtain necessary resources and enact change in some facilities
	*Available resources*	Mixed	Facilitator: Free provision of HIV testing kits by DOHS allowed most clinics to have all necessary resources Barrier: Clinics that were not able to obtain test kits noted this as crucial barrier to successful HTC
**SAIA intervention characteristics**	**Adaptability**	None noted	No clinics reported adapting SAIA
	**Complexity**	Facilitator	All responding clinics reported SAIA to be easy to learn and implement
	**Design quality and packaging**	Facilitator	All clinics approved of the SAIA design, and the training and training materials were cited as particularly useful

*Abbreviations*: *HTC* HIV testing and counseling, *DOHS* Department of Health Services, *SAIA* systems analysis and implementation approach

#### Networks and communication

All but one clinic had regular management meetings (11; 91.7%). These occurred monthly in 6 clinics (54.5%) and quarterly in 5 clinics (45.5%). In addition to these formal meetings, several clinics reported supplementary meetings including hand-off reports between shifts (*n* = 1), as-needed emergency meetings (*n* = 1), and one-on-one meetings (*n* = 1). Clinics also reported sharing information through both formal and informal verbal or written communication. Informal methods of communication included WhatsApp or text messages between clinic staff (*n* = 3, 33%), phone calls (*n* = 2, 22%), and other verbal communications (*n* = 3, 33%). Formal written methods of communication were reported by five (56%) facilities and included emails (*n* = 2) and reports (*n* = 3). Overall, networks and communication appeared to act as a facilitator to HTC implementation, as facilities reported clear communication between team members.

#### Implementation climate

Of the 9 clinics that completed the initial interview, 7 (78%) reported that new changes were embraced at their facility. One in-charge nurse described their facility’s attitude toward change as follows:

“We embrace new ideas. We welcome whenever there is something new. We communicate to each other, we do [continuing medical education (CMEs)] and implement the change."

An in-charge nurse at another clinic described how their fast uptake of HTC during the initial months of the trial demonstrated this commitment to change. However, some interviewees did describe barriers to change within their facilities. Within CFIR, implementation climate is assessed through sub-constructs of tension for change, relative priority of the health topic, goals and priorities, and learning climate. Staff at each clinic were probed about each topic and how they presented as facilitators and barriers toward HTC implementation.

##### Tension for change

When asked about the perceived need to improve HTC at their facility, 78% (*n* = 7) facilities interviewed identified a strong need. One clinic manager specified that unknown HIV status is common among their clientele:

“It’s important for clients to be tested before the [family planning] service is offered. Most don’t know their status and those who turn positive are linked to care.”

One clinic manager specified that the risk of transmitting HIV during pregnancy was a primary factor in this need. A lab technician at another facility recognized that they had a gap in the delivery of HTC, and therefore, improvement was needed. Overall, this perceived need appeared to act as a motivator to conducting HTC, facilitating successful implementation (Table 2).

Not all clinics reported a need for improvement in HTC services. Of the two (22%) clinics that did not perceive this need, a clinic manager at one suggested that they would still be willing to make the change if they had sufficient staff to implement it. A nurse at the other clinic suggested that the change was not needed, and other barriers, such as the time required to make changes, could inhibit implementation.

##### Relative priority

Relative to other initiatives, four (44%) clinics identified HTC improvement as a top priority at their facility. These clinics specified that counselling on HIV (*n* = 3, 33%) and family planning clients knowing their HIV status (*n* = 1, 11%) were their main priority. Other high priorities included provision of family planning products and services (*n* = 4, 44%) and health education (*n* = 1, 11%).

##### Goals and feedback

Interviewees expressed that their facilities had clear goals that were either set internally or by the Mombasa County DOHS. The primary goals included provision of family planning services and HTC. Some clinics identified additional objectives linked to formal initiatives, such as “The Challenge Initiative” targeting uptake of family planning services [24] or Ministry of Health (MOH) efforts to achieve the UNAIDS 90–90-90 goals. Others described more general goals set within clinics, such as ensuring that all MOH programs are implemented at their clinic. Of the clinic staff members that responded to this prompt, all reported that feedback is given to staff members, either internally or through the DOHS. Interviewees identified numerous ways that progress is tracked, and feedback is provided, including through supervision, reports, and review meetings. One clinic manager noted that the process of setting goals and tracking progress with the DOHS can directly help facilities to achieve their goals, as it can result in staff being sent for trainings or clinics being provided with commodities if those are a barrier to success. Overall, clear mechanisms for goal setting and progress tracking appeared to facilitate uptake of new initiatives (Table 2).

##### Learning climate

To assess learning climate, interviewees were asked to report on their problem-solving process within clinics. Of the 9 staff members interviewed, three (33%) described a group consensus approach to initial problem solving, five (56%) described a top-down process where solutions are generated by management, and one (11%) described a formal problem-solving team. Group consensus appeared to facilitate implementation of HTC. Those who described a group consensus approach suggested that staff are involved in the change process, feel empowered to try new things, and generate solutions through discussion. One nurse in-charge noted:

“If there is a problem, we sit and talk about the challenges then we see how best we can solve. Most of the staff involved participate in the process.”

In settings with a top-down approach to problem solving, staff were less involved in solution generation. Often, just one or two individuals were responsible for fixing a problem, and this was presented as a potential barrier to quickly implementing change. Finally, one lab technician described a work improvement team, whose sole function was to identify a problem with their workflow and provide solutions. This formal approach to problem-solving facilitated the implementation of changes within the clinic.

#### Readiness for implementation

Overall, clinic staff expressed a commitment to implementation, largely due to recognizing the need to improve rates of HIV testing among FP clients within their facilities. One lab technician stated:

“We still have a big gap to fill, we still need to pull up our socks.”

Within the CFIR, readiness for implementation is assessed through sub-constructs including leadership engagement and availability of resources. Once again, each sub-construct was assessed in specific probes to clinic staff to understand how they impacted HTC.

##### Leadership engagement

The Mombasa County DOHS was an important source of support for all clinics and facilitated HTC provision in clinics (Table 2). Interviewees identified several ways in which they rely on the DOHS for support, including staffing (*n* = 4, 44%), training and education (*n* = 5, 56%), provision of tools and commodities (*n* = 6, 67%), creating community awareness (*n* = 2, 22%), and follow-up supervision and feedback (*n* = 3, 33%). In addition to external support from the DOHS, interviewees reported strong internal engagement from facility leadership. Clinics that did not report strong leadership engagement suggested that this was a barrier to decision making and acquiring resources, with one nurse stating:

“Admin [leadership] needs to put more effort. We have mentioned about getting [HIV testing] kits but takes forever to make decisions.”

##### Available resources

The county-provided commodities include HIV test kits for testing within family planning clinics. These are provided to clinics at no cost. Despite this, availability of resources was identified as a barrier in three of twelve intervention clinics. Among the three clinics identifying commodities as a barrier, two reported that they had been out of test kits for more than one month, and one responded that they had not yet ordered test kits. All remaining clinics reported that they never run out of HIV testing kits.

##### Summary of inner setting impact on HTC

Taken together, these findings demonstrate that the majority of clinics had a positive implementation climate, engaged leadership, and access to resources. While not all clinics identified HTC as a top priority within their clinic, most reported a strong culture of embracing change and recognition of the importance of HIV testing within FP clinics.

### Intervention characteristics

Data on the study outcomes were collected during the stage 2 interviews, which were conducted with intervention clinic staff after SAIA had been implemented. Seven interviews were completed to evaluate SAIA as an implementation strategy, assessing the CFIR constructs of adaptability, complexity, design quality, and packaging. Acceptability, feasibility, and appropriateness from Proctor’s Implementation Outcomes Framework [17] were assessed using Weiner et al.’s psychometrically validated assessment tools [18]. Staff participating in interviews included in-charge nurses (*n* = 5), a nurse (*n* = 1), and a lab technician (*n* = 1).

#### Adaptability

No interviewee identified any adaptations to SAIA that were made. Some interviews described what micro-interventions or adaptations they made in their clinic to improve HTC, but none described adaptations specific to the five-step SAIA process.

#### Complexity

Overall, SAIA was not thought to be complex, but rather just an extension of the work that is already expected of clinical staff. One in-charge nurse at a clinic stated:

“It was not hard. It is everyday work. Now we are doing what is expected of us. The guidelines say we counsel and test. Most people are not doing that. SAIA helped reach testing targets.”

The low complexity of SAIA appeared to act as a facilitator toward implementation. Initially, some clinic staff were apprehensive that implementing SAIA was going to create extra work, but found this was not the case once they started. In charge nurses at two clinics stated:

“When you hear about SAIA, you think it’s big. It’s not. Just knowing your work and doing it for better outcomes.”

“At first thought it was going to be a lot, but it didn't affect work. It worked well.”

#### Design quality and packaging

The design of SAIA was also identified as a facilitator to implementation. Many interviewees praised the SAIA training, and in particular, the training booklet that was distributed. One in-charge nurse felt that the SAIA training was practical and that the sequential process flow mapping was informative:

“The training was very practical. Seeing how a patient moves from entering clinic to leaving was an eye opener. Also, at first, we had issues with documentation which SAIA was able to address. I still have a training book at my desk.”

#### Acceptability

Intervention outcomes were assessed using Weiner et al.’s assessment tools combined with semi-structure interview questions [18]. SAIA was rated as very acceptable with a median Likert score of 5 (IQR 5–5). One in-charge nurse said:

“SAIA was accepted, and we are in it now."

Another in-charge nurse went so far as to say all facilities should implement SAIA for other health outcomes as well, not just in the FP clinic. They stated:

“Staff are dedicated to make SAIA work and with support of subcounty reproductive health/STI [officers]. I think all facilities in county should have SAIA and improve all health outcomes, not just FP clinic."

Clinic staff also liked that SAIA facilitated generation of solutions to problems by the primary healthcare providers in the clinic. This was not a top-down approach. One in-charge nurse stated:

“Coming up with own solutions. This was impressive. They were solutions by us, not someone in charge at the top, but us. We attended training. Most times it's only management [who attend trainings] who don't even know the work."

A lab technician said that they will continue to implement SAIA even though the study is over because it was so helpful and there was a sense of agency:

"The solutions were from us...we shall continue doing [SAIA] even as you say the study is over."

#### Appropriateness

Interviewees felt that SAIA was appropriate in their clinical and situational context and provided benefit to their staff and clients. Respondents rated SAIA as very appropriate with a median Likert score of 5 (IQR 5–5). There were no parts of SAIA that were felt to be ill-suited or not fitting. A lab technician said:

“[SAIA] works for us […] Knowing the problems and solving them really helped us.”

An in-charge nurse felt that SAIA worked very well for them and helped them address the barriers to HTC within their specific facility, which included their practice of charging clients for HIV tests. This clinic ultimately waived the fee for HIV testing to eliminate this barrier to HTC:

“SAIA is the best. We were charging women [for HIV tests] before and not testing any FP women.”

#### Feasibility

Respondents rated SAIA as very feasible and doable in their environment and setting with a median Likert score of 5 (IQR 5–5). An in-charge nurse stated:

“All is well with SAIA. It is easy. Document, count, and see results.”

Another in-charge nurse strongly emphasized how SAIA was doable and easy to implement:

“All worked, especially the monitoring you guys were doing. Training was very important in getting people started and knowing what to expect.”

Finally, one nurse liked that SAIA helps clinic staff generate solutions on their own with “available limited resources,” highlighting that SAIA works within a given system to facilitate improvements that are doable in the hands of the staff.

##### Outcome summary

Stage 2 interviews highlighted the high acceptability of SAIA, usefulness of the SAIA training, lack of complexity, and good fit within existing clinic processes. In particular, staff praised the model that SAIA uses in which intervention ideas are generated and implemented by front-line healthcare workers within the clinic. This agency was empowering and generated ideas and solutions that were appropriate and feasible.

## Discussion

This analysis used surveys and interviews with clinic staff to evaluate the use of SAIA in FP clinics in Mombasa County, Kenya, as an effective strategy to increase HTC [5]. The results suggest that SAIA was highly feasible, acceptable, and appropriate at these clinics. The relative simplicity of the intervention and the quality of training were important facilitators of intervention success. Implementation of HTC was facilitated by strong communication structures within the clinics, a climate that embraced change, and leadership engagement. Lack of HIV testing supplies was identified as a barrier at some clinics, and low leadership engagement was identified as a barrier to implementation at a minority of clinics. Overall, clinic staff were satisfied with SAIA and believed it was effective at increasing HTC within their facilities.

The combined use of CFIR and Proctor’s IOF in evaluating SAIA provided a comprehensive framework to examine the multi-level context that impacted implementation [15]. While CFIR is increasingly being used as an evaluative framework in low- and middle-income countries (LMICs) [25], few studies have used it to assess outcomes of a SAIA intervention [9]. In one study, CFIR was used to evaluate SAIA as an implementation strategy for optimizing PMTCT of HIV services in Mozambique, Kenya, and Cote d’Ivoire comparing low and high performing facilities. The authors found that *networks and communication*, *available resources*, *external change agents*, *executing*, and *reflecting and evaluating* were strongly associated with high performing clinics. While clinics in our study did not differ enough in study outcomes to compare high and low performers, there are parallels between the distinguishing characteristics identified in the evaluation of SAIA for PMTCT and the facilitators identified in our study. In both cases, strong communication and availability of key resources were identified as key drivers of success. In addition, engagement of leadership was identified as a crucial component of implementation success in both studies. The constructs associated with implementation success in both studies align with characteristics related to successful implementation in previous research in low-resource settings [15]. A recent systematic review of the use of CFIR in LMICs found that the CFIR constructs “*complexity*” (e.g., the perceived difficulty of the intervention) and “*networks and communication*” (e.g., the nature and quality of formal and informal communication within an organization) were the most commonly used constructs in these settings. Further, all the constructs identified as important in our study were identified as compatible for use in global settings, suggesting these constructs are applicable to broader settings in LMICs.

This research provides early evidence of the mechanisms of action through which SAIA works to impact care delivery. The field of implementation science is in the early stages of investigating how and why implementation strategies produce desired outcomes [26]. Understanding these mechanisms is crucial to determine selection and application of implementation strategies to best address barriers. Important mechanisms of SAIA have been hypothesized across multiple studies [27]. SAIA is used as a tool for healthcare teams to identify and prioritize problems or bottlenecks, and then implement and evaluate changes to address those bottlenecks [10, 14]. Previous SAIA studies have shown that this method leads to better outcomes by improving communication, consensus decision-making, and accountability across staff within a care cascade [13, 14]. In our study, removing barriers to inner setting characteristics was the focus of many micro-interventions, as outlined in the previously published trial results [5]. For example, lack of clinic space and availability of HIV test kits were addressed in several SAIA cycles. Successful implementation of micro-interventions to address these barriers ultimately led to an increase in HTC delivery in the intervention clinics. The outcomes of this study provide additional data on the salient action mechanisms through which SAIA operates, and future research could build on the evidence generated in our study to examine how mechanisms differ when applied to varying contexts.

Our findings could be used to directly inform implementation of future SAIA applications. The mechanisms of action identified here could provide a beginning framework for clinics to assess barriers that might impact implementation at their clinic, and ensure these barriers are addressed either before study start or through micro-interventions in the course of the study. For example, we found that low leadership engagement was a barrier to successfully enacting change, so facilities may focus on engagement of leadership early in an intervention. Another example would be assessing the degree to which availability of resources is a barrier at each facility. While most FP clinics had reliable access to HIV test kits, this was a crucial barrier to uptake of HTC for those that did not. Given these findings, future studies using SAIA could target availability of resources and leadership engagement as key first components in SAIA implementation to improve efficiency and increase the likelihood of success.

This evaluation of SAIA had some notable strengths. It incorporated interviews during initial SAIA implementation to understand the FP clinic landscape. Post-SAIA implementation interviews collected feedback about SAIA from the main SAIA implementers within each clinic. Data were captured from the inner setting and intervention characteristics to provide a broad overview of where SAIA was implemented, and validated questionnaires were used to assess how acceptable, appropriate, and feasible it was. This adds to the growing literature around SAIA as a useful and acceptable implementation strategy in LMICs.

This study had several limitations. Past studies using CFIR to evaluate implementation strategies have identified distinguishing features between high-and low-performing clinics. This was not possible in the present study, as there was not enough of a difference in study outcomes between high-and low-performing clinics. While it was outside the scope of this current study, comparing how baseline and end of study inner setting characteristics varied between high and low performing sites may elucidate the underlying mechanisms and barriers to SAIA’s implementation. Furthermore, it was not possible to interview at least one staff member from all intervention clinics following the intervention due to interruptions caused by the COVID-19 pandemic. This may have biased the results if the clinics that were interviewed were either more willing to participate because of favorable opinions about SAIA or had more resources to remain open during the pandemic. However, quantitative results highlighting SAIA’s effectiveness in increasing HTC in FP clinics further support SAIA’s acceptability, feasibility, and appropriateness even in clinics not being interviewed. Lastly, our sample size of 12 clinics is small and could be seen as a limitation, but this sample represented all intervention clinics in this cluster-randomized trial. One small private clinic did not participate in SAIA interventions or interviews, but participated in the stage one questionnaire and allowed outcome data to be collected. This clinic denied participation due to a perceived lack of interest in HIV testing among their clientele, and ultimately did not provide HIV testing at their clinic during the study period. This example highlights the importance of prioritization and perceived need of an evidence-based intervention in determining the success of an implementation strategy.

Future directions for this work include scaling up SAIA to reach more FP clinics in Mombasa County and expanding SAIA implementation to include downstream steps in the HIV prevention and treatment cascade. Given the high acceptability of SAIA for increasing HTC in FP clinics, this implementation strategy seems likely to have broad stakeholder and clinic staff buy-in for the next phase of this research.

## Conclusion

In conclusion, SAIA was implemented in FP clinics of different sizes, capacities for change, and management support. SAIA was acceptable, appropriate, feasible, and not complex. The agency that clinic staff felt in proposing their own interventions and implementing them was an important contributor to SAIA’s success. SAIA’s acceptability, feasibility, and low complexity coupled with clinic staff’s agency will be crucial when led by non-research personnel and scaled up to more facilities in future work.

## Data Availability

This study was conducted with approval from the Kenyatta National Hospital—University of Nairobi Ethics and Research Committee (KNH-UON ERC), which requires that we release data from Kenyan studies (including de-identified data) only after they have provided their written approval for additional analyses. As such, data for this study will be available from the authors upon request, with written approval for the proposed analysis from the KNH/UON ERC. Their application forms and guidelines can be accessed at https://erc.uonbi.ac.ke/. Please contact The Principal of the KNH-UON ERC at principal-cae@uonbi.ac.ke.
